# *Burkholderia pseudomallei* Is Genetically Diverse in Agricultural Land in Northeast Thailand

**DOI:** 10.1371/journal.pntd.0000496

**Published:** 2009-08-04

**Authors:** Vanaporn Wuthiekanun, Direk Limmathurotsakul, Narisara Chantratita, Edward J. Feil, Nicholas P. J. Day, Sharon J. Peacock

**Affiliations:** 1 Mahidol-Oxford Tropical Medicine Research Unit, Faculty of Tropical Medicine, Mahidol University, Bangkok, Thailand; 2 Department of Biology and Biochemistry, University of Bath, Bath, United Kingdom; 3 Center for Clinical Vaccinology and Tropical Medicine, Nuffield Department of Clinical Medicine, University of Oxford, Churchill Hospital, Oxford, United Kingdom; 4 Department of Medicine, University of Cambridge, Addenbrooke's Hospital, Cambridge, United Kingdom; McGill University Health Centre, Canada

## Abstract

**Background:**

The soil-dwelling Gram-negative bacterium *Burkholderia pseudomallei* is the cause of melioidosis. Extreme structuring of genotype and genotypic frequency has been demonstrated for *B. pseudomallei* in uncultivated land, but its distribution and genetic diversity in agricultural land where most human infections are probably acquired is not well defined.

**Methods:**

Fixed-interval soil sampling was performed in a rice paddy in northeast Thailand in which 100 grams of soil was sampled at a depth of 30 cm from 10×10 sampling points each measuring 2.5 m by 2.5 m. Soil was cultured for the presence of *B. pseudomallei* and genotyping of colonies present on primary culture plates was performed using a combination of pulsed-field gel electrophoresis (PFGE) and multilocus sequence typing (MLST).

**Principal Findings:**

*B. pseudomallei* was cultured from 28/100 samples. Genotyping of 630 primary colonies drawn from 11 sampling points demonstrated 10 PFGE banding pattern types, which on MLST were resolved into 7 sequence types (ST). Overlap of genotypes was observed more often between sampling points that were closely positioned. Two sampling points contained mixed *B. pseudomallei* genotypes, each with a numerically dominant genotype and one or more additional genotypes present as minority populations.

**Conclusions:**

Genetic diversity and structuring of *B. pseudomallei* exists despite the effects of flooding and the physical and chemical processes associated with farming. These findings form an important baseline for future studies of environmental *B. pseudomallei*.

## Author Summary

*Burkholderia pseudomallei* is the cause of melioidosis, a serious human infection most commonly diagnosed in southeast Asia and northern Australia. The organism lives in the soil in a specific geographical distribution and infection results from bacterial inoculation, inhalation or ingestion. The purpose of this study was to define the distribution and genetic diversity of *B. pseudomallei* in agricultural land where most human infections probably occur. We performed soil sampling and culture for the presence of *B. pseudomallei* in 100 equally spaced points within a rice paddy in northeast Thailand, and undertook genotyping of primary culture plate colonies from 11 sampling points. We identified 7 different genotypes, with relatively limited overlap between different sampling points. Two samples contained more than one *B. pseudomallei* genotype, in which a numerically dominant genotype coexisted with one or more additional genotypes present as a minority population. We conclude that genetic diversity and structuring of *B. pseudomallei* exists despite the effects of flooding and the physical and chemical processes associated with farming. These findings inform future efforts to define *B. pseudomallei* in the environment, and should be considered during the design stage of studies comparing *B. pseudomallei* isolated from the environment and from patients with invasive disease.

## Introduction

The Gram-negative bacterium *Burkholderia pseudomallei* is the cause of melioidosis, a serious human infection with a mortality rate as high as 50% in Thailand and 20% in Australia [1],[2]. The organism is found in the environment across much of southeast Asia and northern Australia where infection may be acquired following bacterial inoculation and contamination of wounds, inhalation or ingestion [1],[2]. Environmental sampling to detect the presence of *B. pseudomallei* serves to map its distribution and define geographical regions of risk to humans and livestock. Bacterial genotyping of isolates from the environment and cases of disease is an essential component of outbreak investigations to link isolates to a common contaminated source [3]–[5], and has also been performed to provide insights into the relationship between pathogenic *B. pseudomallei* and the environmental population from which they are drawn [6]. These applications require sampling strategies that detect an unbiased and true representation of the bacterial population under study.

A previous study conducted by us to define the genetic diversity of *B. pseudomallei* isolated from uncultivated land in northeast Thailand demonstrated that genetic heterogeneity both within a single soil sample and between samples taken within a geographically restricted sampling site were much greater than recognised previously [7]. Genotyping of 200 primary *B. pseudomallei* colonies at each of three sampling points 7.6 to 13.3 meters apart demonstrated that each point contained a predominant *B. pseudomallei* multilocus sequencing typing (MLST) sequence type (ST) together with 2 or 3 additional STs present as minority populations, and that genotypes were rarely shared between the three sampling points [7]. These findings have major implications for genotyping studies of environmental *B. pseudomallei*, and indicate that delineation of genotypes present even in a small area of the natural environment requires sampling of multiple primary *B. pseudomallei* colonies from multiple points. Although these studies shed light on the structure of natural populations, it is not clear whether these results from uncultivated land are translatable to agricultural land where the majority of human exposure and disease acquisition probably occurs. Anthropogenic disturbance resulting from ploughing, planting, the presence of rice, flooding, the application of chemical fertilizers and pesticides and rice stubble burning could have a major impact on *B. pseudomallei* presence, genotypes and distribution of genotypes. The objective of the study described here was to undertake a detailed evaluation of *B. pseudomallei* in a rice paddy, and to compare and contrast the results to our previous data from uncultivated land.

## Methods

### Study site

Soil sampling was performed on the 9th May 2007 (the start of the rainy season) in a single rice paddy situated in Amphoe Lao Sua Kok, Ubon Ratchathani, northeast Thailand. This is a rural rice-growing region where buffalo and hand-held implements are used for ploughing. The site had been used for rice cultivation for at least 25 years, and was isolated by raised earth walkways on three sides and by a dirt road on the fourth side. The soil type was sandy loam and was moist but not wet during sampling. The paddy had been ploughed in the preceding two weeks but not planted. The site was divided into a grid system using string and wooden stakes in which 10×10 sampling points each measuring 2.5 m by 2.5 m were defined. Standing on the bank directly opposite the dirt road, these were numerically and alphabetically referenced from left to right into ten rows of ten squares (grid reference A1-10 top row, B1-10 second row, and so on).

### Soil sampling, culture and bacterial identification

Soil was removed from a depth of 30 cm at the centre of each sampling point and 100 grams from each put into a pre-labelled plastic bag placed on weighing scales. Samples were stored at ambient temperature out of direct sunlight until transported to the laboratory for same day processing. Sampling utensils were cleaned between each use by being rinsed with bottled water to removed visible debris, cleaned with 70% ethanol and air dried prior to next use. Soil was cultured for the presence of *B. pseudomallei* by adding 100 ml of sterile water to each bag, mixing well and leaving overnight to sediment, and then transferring the upper liquid layer to a sterile plastic container prior to culture. Aliquots (2×10 µl, 2×100 µl and 1×500 µl) were each spread plated onto Ashdown selective agar plates, which were incubated at 40°C in air and inspected daily for 4 days. A further 1 ml of supernatant was added to 9 ml of selective enrichment broth consisting of threonine-basal salt plus colistin (TBSS-C50 broth) and incubated at 40°C in air for 48 h, after which 10 µl of surface liquid was plated onto Ashdown agar which was incubated and inspected as before. Colonies of *B. pseudomallei* were initially identified on the basis of colony morphology appearance. This included the characteristic colony morphology (purple, flat, dry and wrinkled) together with 6 additional colony morphotypes, as described previously [8]. Colour photographs of these colony types were available throughout the study to aid identification. Colonies suspected to be *B. pseudomallei* were tested using the oxidase test, and positive colonies confirmed as *B. pseudomallei* using a highly specific latex agglutination test (positive for *B. pseudomallei* but negative for *B. thailandensis*) [9],[10].

### Genotyping of *B. pseudomallei*

*B. pseudomallei* genotyping of individual colonies was performed using a combination of pulsed-field gel electrophoresis (PFGE) and MLST. Individual primary colonies were subcultured onto Columbia agar and incubated for 48 h at 37°C in air prior to harvest and PFGE using *Spe*I, as previously described [11], and the banding patterns analysed using the BioNumerics software version 2.5 (Applied Maths, Belgium). Isolates with identical PFGE banding patterns were regarded as genetically indistinguishable, but isolates with one or more bands different were defined as putatively different and given a different banding pattern number. One bacterial representative of each banding pattern type was further examined using MLST, as described previously [6]. The alleles at each of the loci were assigned and sequence type defined using the *B. pseudomallei* MLST website (http://bpseudomallei.mlst.net). Splits decomposition analysis was carried out on concatenated sequences using SplitsTree 4 [12].

## Results

A total of 28/100 soil samples were culture positive for *B. pseudomallei* on direct plating onto Ashdown agar; selective enrichment broth did not identify additional samples positive for *B. pseudomallei*. A single colony morphotype (Type I, the commonly recognized ‘cornflower head’ morphology on Ashdown agar) was identified in all positive samples, with no variability in *B. pseudomallei* colony morphology appearance within or between samples.

**Figure 1:**
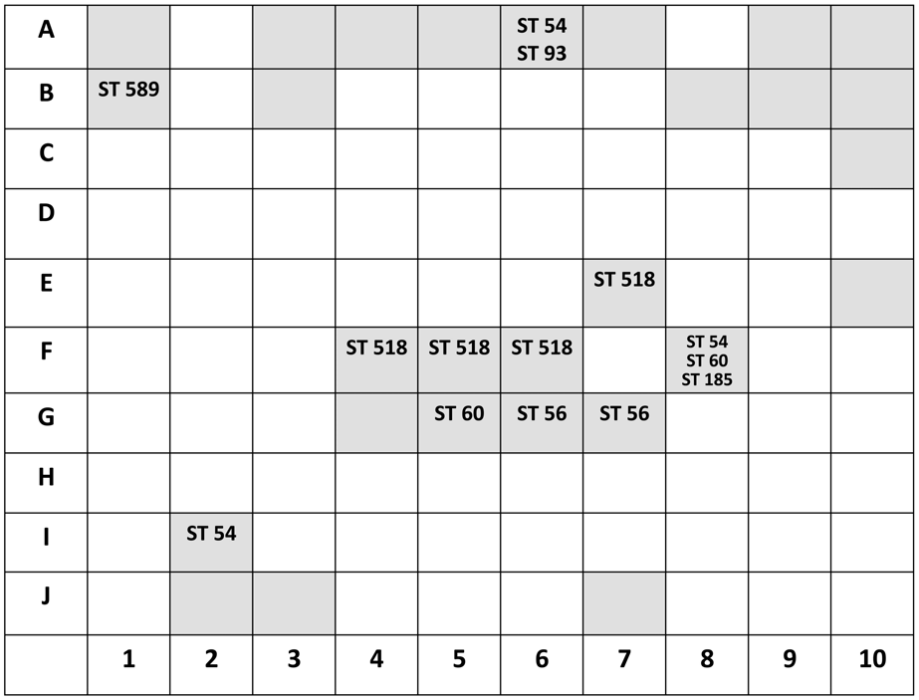
The presence of *B. pseudomallei* in 100 equally spaced sampling points measuring 2.5 m×2.5 m. Absence of *B. pseudomallei* at a sampling point is denoted by a blank white box. Presence of *B. pseudomallei* at a sampling site is denoted by a grey box, superimposed with sequence type (ST) data where genotyping was performed. The number of colonies genotyped was 60 for all points with typing data shown, with the exception of E7 and F6 which only contained 45 colonies on the initial primary plates.

Genetic variability of *B. pseudomallei* was defined both within an individual soil sample and between sampling points. Genetic variability within a single sample was defined by genotyping 60 separate colonies picked from primary culture plates for each of 11 sampling points with the exception of E7 and F6, which only contained 45 colonies on the initial primary plates. This gave a total of 630 primary plate colonies for genotyping. Genetic variability between sampling points was evaluated at close range (defined as sampling points within 5 meters of F6) for 7 points positive for *B. pseudomallei* (E7, F4, F5, F8, G5, G6, and G7, see Figure 1). Genetic variability between sampling points at longer range was evaluated for 3 points containing *B. pseudomallei* that were chosen at random outside the 5-meter range from F6 (A6, B1 and I2).

PFGE of the 630 primary colonies identified ten different PFGE banding pattern types (PTs) (Table 1). MLST performed on a single representative of each of the 10 PTs identified seven STs. Analysis of genetic variability within individual samples demonstrated that 9 of 11 sampling points contained a single ST, one point (A6) contained two STs, and one (F8) contained three STs (Figure 1). Of the two sampling points containing mixed *B. pseudomallei* genotypes, both contained one genotype that was numerically dominant (ST 93 at sampling point A6 and ST 54 at sampling point F8, Table 1). Several STs were shared across closely positioned samples (Figure 1). For example, ST 518 was present at E7, F4, F5 and F6, and ST 56 was present at G6 and G7. ST 54 was widely distributed across the field, being identified at A6, F8 and I2. The genotypes at the more distantly positioned sampling points were diverse and different from genotypes clustered around point F6 (Figure 1).

**Table 1:** Genotyping of *B. pseudomallei* from eleven sampling points.

ST	PFGE type (PT)	MLST allelic profile							Grid reference and number of colonies per point										
		*ace*	*gltB*	*gmhD*	*lepA*	*lipA*	*nark*	*ndh*	A6	B1	E7	F4	F5	F6	F8	G5	G6	G7	I2
ST 518	PT A_1_	1	1	13	2	1	1	1			45	60	60	44					
	PT A_2_													1					
ST 60	PT B_1_	3	1	12	1	1	3	1							1	60			
ST 56	PT C_1_	3	1	4	1	1	4	1									60	59	
	PT C_2_																	1	
ST 54	PT D_1_	3	1	3	3	1	2	1	1						42				
	PT D_2_																		60
ST 185	PT E_1_	1	4	2	2	1	4	1							17				
ST 93	PT F_1_	1	1	2	1	1	4	1	59										
ST 589	PT G_1_	3	1	11	1	1	1	1		60									

A total of 60 colonies were picked for genotyping from primary culture plates for all sampling points except for E7 and F6, for which the total was 45 per point. PT C_2_ and PT D_2_ differed by one band from PT C_1_ and PT D_1_, respectively. A1 and A2 differed by 7 bands.

**Figure 2:**
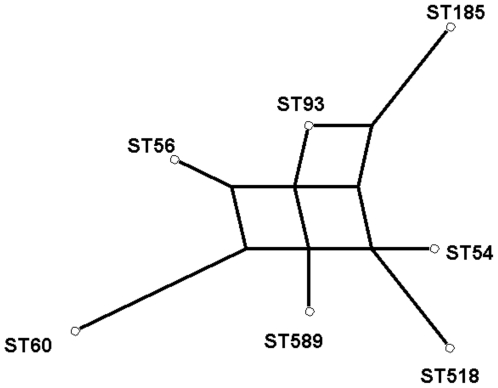
Splits decomposition based on the concatenated sequences of the seven STs identified from 11 sampling points. This was carried out using SplitsTree 4 (splitstree.org) using the default settings. The analysis suggests that the STs are approximately equidistant from each other, and the high level of reticulation is consistent with free recombination.

The genetic relationships between *B. pseudomallei* STs isolated from the paddy was considered in relation to allelic profiles and concatenated sequences. The average number of allelic differences between pairs of STs was 3.28. In terms of allelic profile, ST185 was marginally the most diverged genotype, differing on average by 4 alleles from the other 6 STs, although this was similar to the equivalent figures for the other STs (2.83–3.66). Therefore, no STs are highly divergent from all the others, and no pairs or clusters of highly related STs are apparent. Such a pattern is consistent with previous studies demonstrating high rates of recombination and low levels of linkage within the *Burkholderia* population [6],[13]. Given this, we would not expect to find a strong phylogenetic signal in these data, and this is supported by splits decomposition analysis which reveals extensive reticulation reflecting conflicts in the data consistent with recombination (Figure 2).

The STs identified from soil were also compared with those listed by the *B. pseudomallei* MLST website. ST 54, ST 56, ST 60 and ST 93 have been isolated previously from both the environment in Thailand and from patients with melioidosis in Thailand (ST 54, ST 56 and ST 60), or the United States (ST 93), which we presume was acquired elsewhere. ST 185 and ST 518 have not been isolated previously from the environment but were isolated from a Thai patient with melioidosis (ST 185) or an animal in the USA (ST 518), which we again presume was imported. ST 589 has not been isolated previously and is unique to this study.

Three PTs contained banding variability (Table 1). PT A contained one colony (PT A_2_) out of a total of 210 colonies with seven bands difference compared with the predominant pattern of PT A_1_, and PT C contained one colony (PT C_2_) out of a total of 120 colonies with one band difference compared with the predominant pattern of PT C_1_. As the rare variants PT A_2_ and PT C_2_ were found in the same sites as their parent clones, it is likely that these have diversified *in situ*. In contrast, PT D had two banding patterns consisting of a single band difference that were completely segregated into two different sampling points, which is consistent with two independent introduction events.

## Discussion

Rice paddies are subjected to anthropogenic disturbance via multiple physical and chemical events that might be predicted to lead to homogeneity in both the physical distribution and genotype of *B. pseudomallei* across our sampling site. In particular, ploughing and flooding may result in mixing of organisms and homogenization of genotypes. Despite this, *B. pseudomallei* was variably present across the rice paddy and genetic diversity was observed across the sampling site overall and within some of the individual samples. This has two major implications for future studies and for our understanding of disease. First, agricultural workers may encounter a variable number of genotypes even within a restricted area of land. This sets the scene for exposure to a potentially heterogeneous population of isolates, some of which could have a higher potential to cause disease than others. Although this suggestion is speculative at the present time, variability in genome content between strains is high [14], and we propose that this may influence both the ability to infect the human host and the virulence of the organism once invasion has occurred. Consistent with this are the observations that virulence of different strains show marked differences in experimental animal models [15],[16], and that simultaneous infection with more than one strain of *B. pseudomallei* is uncommon in humans (1.5% of cases) [17]. The second implication for our findings is that the accuracy of genotyping of *B. pseudomallei* in agricultural land will depend on testing multiple colonies from multiple sampling points. We have yet to define the full extent of diversity within a restricted sampling area, and we predict that diversity will be greater than that defined thus far from the limited number of samples tested here. Studies that aim to compare isolates from the environment with isolates causing invasive disease will need to take account of the sampling strategy of the environmental collection under test and consider whether this will provide an accurate representation of the population from which it was drawn.

The study reported here was undertaken in a paddy field situated 19 km southwest of a sampling site used previously to defined *B. pseudomallei* distribution and genetic diversity in an area of uncultivated land covered with low lying scrub [7]. One hundred samples were cultured from this site using fixed-interval sampling and comparable culture methodology, with genotyping being performed on 600 colonies from 3 sampling points (200 from each) [7]. The weather conditions and wetness of the soil on the day of sampling and the soil type were comparable between the two sites, although sampling was performed in different calendar months. These two datasets provide the opportunity to compare and contrast the results arising from the two sites.

A striking difference between rice paddy and uncultivated land was the number of sampling points that were positive for *B. pseudomallei* (28% for rice paddy compared with 80% for uncultivated land, p<0.001 Chi-square test). A possible explanation is that chemicals and/or fertilisers used in the rice paddy had an adverse effect on *B. pseudomallei* survival. These might also select for some genotypes over others and so lower the overall diversity. In the last 15 years, no pesticides have been applied to the paddy field studied here and inorganic fertiliser has been applied three times per year between rice planting and harvest. An alternative explanation is that variable positivity between different sampling sites represents larger-scale geographical variation. Other factors include possible variability in the content of water and organic matter between the two sites, and different sampling dates (May 2007 and September 2005). These two studies are the first of their kind to undertake fine-scale mapping of *B. pseudomallei* positivity using fixed-interval sampling over a restricted area, and further studies are required to determine how much variation can be accounted for by natural variation and how much is due to farming practices. We hypothesize that positivity rates will be influenced by both factors. Estimates of variability are particularly important when sampling geographical areas where the bioburden of *B. pseudomallei* in the environment is low. Under such circumstances, the probability of a false negative culture result is more likely to occur, and this will be compounded by natural variation in positivity rate.

The number of STs present in a single soil sample was lower in the rice paddy compared with uncultivated land. Of 11 rice paddy sites, 9 contained a single ST while all three uncultivated land soil samples examined contained three or four STs. One explanation for this is that only 45 or 60 colonies were typed from each of 11 rice paddy samples, which could fail to detect genotypes present at a frequency of less than 1 in 60. In our previous study we found that the lowest frequency with which a given genotype was detected was 9 in 200 colonies. From this we estimated based on the exact 95% binomal confidence interval for this genotype that approximately 50 colonies from a single site would provide an 85% probability of detecting a genotype present at a sampling point at a frequency of 2%. This calculation was the rationale for genotyping 60 colonies from each sampling site in the rice paddy, where we found the lowest proportion for a given genotype to be 1 in 60 (points A6 and F8). The possibility remains that *B. pseudomallei* may be present as a proportion of the population that falls below this level of detection, but genotyping on a larger scale becomes impractical using the technology described here and would require alternative approaches. The finding that *B. pseudomallei* genotypes were more homogenous within single soil samples from rice paddy may be related to ploughing, which could break down soil macro-aggregates and/or change the ecological environment in such as way that co-existence of multiple *B. pseudomallei* genotypes is no longer sustainable. It is also possible that agricultural practices lead to changes in the community structure of other members of the *Burkholderia* genus, and that this has an effect on the presence and/or diversity of *B. pseudomallei*. This possibility is consistent with the findings of previous studies conducted in regions where *B. pseudomallei* was not present in the environment, in which agricultural management such as fertilization and tillage was shown to alter the community structure of members of the *Burkholderia* genus [18],[19].

A comparison of the *B. pseudomallei* STs identified in the rice paddy and uncultivated land revealed that three STs (ST 60, ST 93 and ST 185) were identified in both sites. Comparison of PFGE banding patterns within each pair of strains demonstrated that the pattern was the same in both sites for ST 185. In uncultivated land, ST 60 and ST 93 had more than one PFGE banding pattern (3 and 2 banding pattern types, respectively). The banding patterns observed for ST 60 and ST 93 from the rice paddy matched one of the patterns observed for each ST in uncultivated land, suggesting that the same clone is present in two different sites. Further studies are required to determine whether some STs are found at a higher frequency in the environment, suggesting that these may have an adaptive fitness advantage. The PFGE data from both sites are indicative of fine-scale geographic structuring within individual clones.

A possible criticism of the typing strategy used in this study is that the assumption that an identical PFGE banding pattern between two or more strains can be taken to infer genetic identity may be incorrect, and that different clones could share the same PFGE banding pattern. We predict based on the high rate of recombination for this organism [6],[20] that the probability of two identical PFGE banding patterns representing different clones of *B. pseudomallei* is low. However, we cannot exclude this possibility and so we considered what effect this putative error would have for our data. We suggest that this would result in an underestimate of genetic diversity, and that the extent of diversity reported here is a minimum estimate of true diversity. The degree of diversity defined here exceeded expectations and previous assumptions for rice paddy, and finding even great diversity would only serve to strength the case for diversity of *B. pseudomallei* in the environment.

A previous study performed in the neighbouring province of Khon Kaen in northeast Thailand in which 68 *B. pseudomallei* colonies were characterised from 19 positive soil samples taken from intervillage roads over a 50 km^2^ region yielded only two STs (ST 70 which is the commonest cause of melioidosis in Thailand, and a novel ST with the allelic profile 3-1-3-2-5-2-1) [21]. It is not clear whether this lack of variation compared to the current study reflects a higher diversity in Ubon Ratchathani, or simply differences in sampling and genotyping strategies. Further studies are required to determine whether our finding of genetic diversity is more widely applicable across the region.

The colony morphology of *B. pseudomallei* isolated from rice paddy was Type I alone, and from uncultivated land was predominantly Type I (76/77 positive primary plate cultures) [7]. Taken together, these studies indicate that Type I appears to be the preferential morphotype for *B. pseudomallei* in the specific environments examined during the two studies. This contrasts with the colony morphology appearance of invasive *B. pseudomallei* in primary culture plates from human samples. A prospective study of 128 unselected patients with 218 samples positive for *B. pseudomallei* demonstrated that Type 1 predominated, but that more than 8% of primary cultures demonstrated colony morphology variability on Ashdown agar [8]. The fitness benefit associated with changing morphology and the possible conditions under which these occur are intriguing questions.

In conclusion, we found that *B. pseudomallei* had a patchy distribution in a rice paddy in northeast Thailand, and have described evidence for genetic diversity and fine-scale genetic structuring in this setting. This contrasted with our expectations that planting and cultivation would lead to homogenous dissemination of a particular bacterial lineage throughout the field. The stability of bacterial distribution and presence of specific genotypes in a single site over time is the subject of on-going studies.
